# Supplementary material for: Tailoring vapor film beneath a Leidenfrost drop
Source: Nat Commun. 2023 May 8;14:2646. doi: 10.1038/s41467-023-38366-z (PMC10167315; doi:10.1038/s41467-023-38366-z)
Supplement: Supplementary file 1 — Supplementary Information [file 41467_2023_38366_MOESM1_ESM.pdf]

Supplementary Information for

Tailoring vapour film beneath a Leidenfrost drop

An Li[1,2], Huizeng Li[1*], Sijia Lyu[3], Zhipeng Zhao[1,2], Zheng Li[1], Kaixuan Li[1,2], Luanluan Xue[1,2], Mingzhu Li[1], Chao Sun[3,4*] & Yanlin Song[1,2*]

[1]Key Laboratory of Green Printing, CAS Research/Education Center for Excellence in Molecular Sciences, Beijing National Laboratory for Molecular Sciences, Institute of Chemistry, Chinese Academy of Sciences, Beijing 100190, P. R. China.

[2]University of Chinese Academy of Sciences, Beijing 100049, P. R. China.

[3]Center for Combustion Energy, Key Laboratory for Thermal Science and Power Engineering of Ministry of Education, Department of Energy and Power Engineering, Tsinghua University, Beijing 100084, P. R. China.

[4]Department of Engineering Mechanics, School of Aerospace Engineering, Tsinghua University, Beijing 100084, P. R. China.

*Correspondence to: ylsong@iccas.ac.cn; chaosun@tsinghua.edu.cn; lihz@iccas.ac.cn.

The Supplementary Information include:

Supplementary Figs. 1 to 11

Supplementary Text

Other Supplementary Information for this manuscript include the following:

Movies 1 to 11 (.mov)

Supplementary Figures

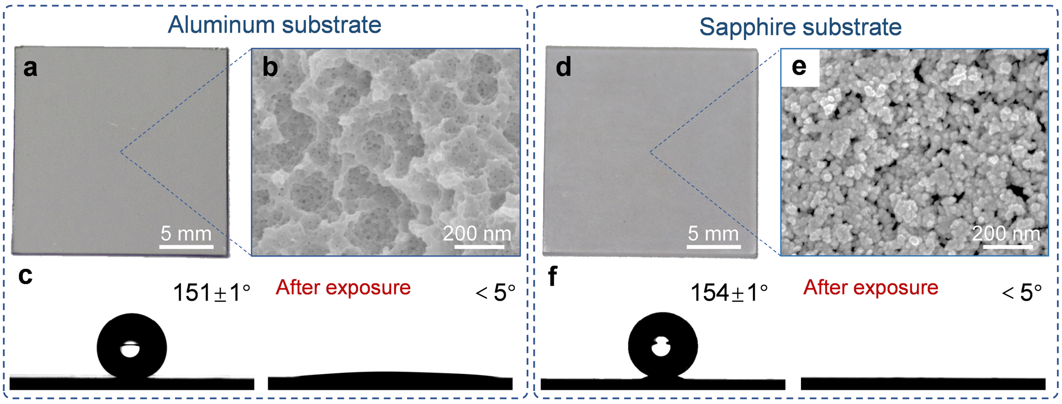

Supplementary Figure 1. Characterizations of the surfaces. a, Photograph of the porous aluminum substrate. **b**, Scanning electron microscope (SEM) image of the porous aluminum substrate. **c**, Contact angles of the substrate before (left image) and after UV exposure (right image). **d-f**, Photograph, SEM image, and contact angles of the sapphire substrate.

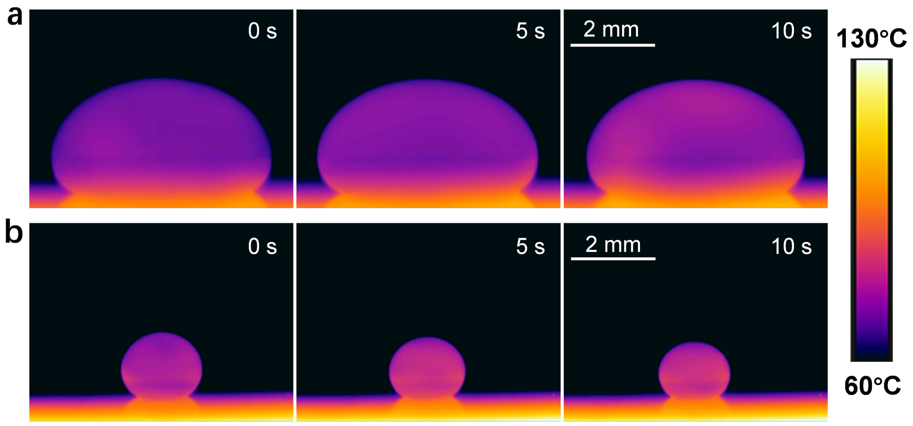

Supplementary Figure 2. Temperature differences of different-sized drops. a, A temperature difference existed in the drop with a radius of 3 mm. **b**, No obvious temperature difference was observed in the small droplet of 1 mm in radius.

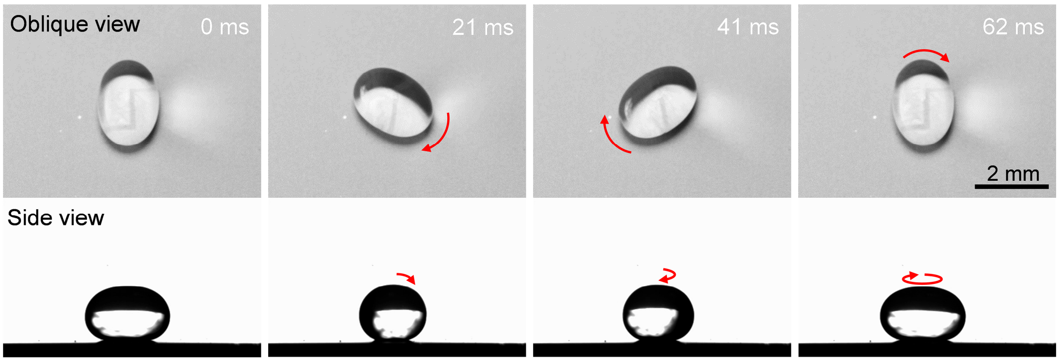

Supplementary Figure 3. Spinning droplet with a radius of 1 mm. The small size droplet can steadily spin on the patterned wettability surface.

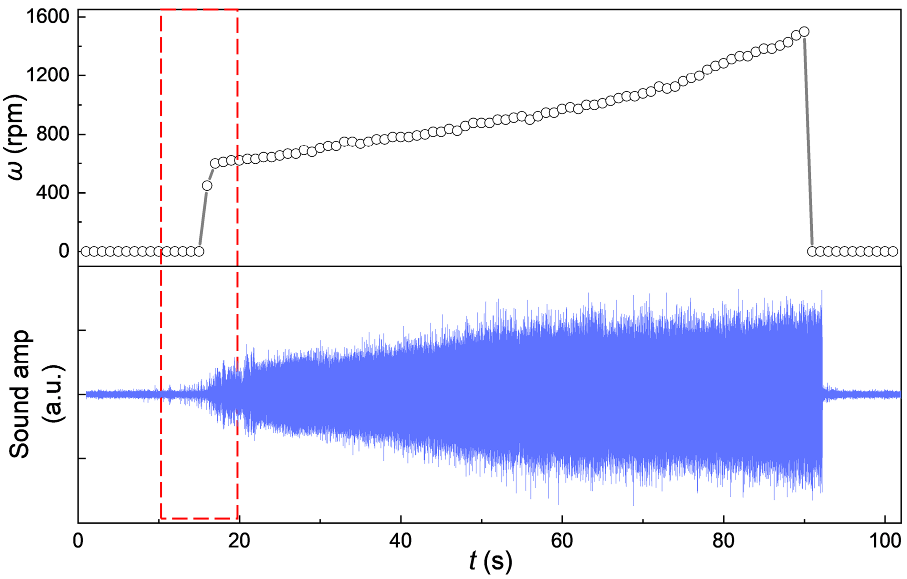

Supplementary Figure 4. The rotational speed *ω* and the sound signal versus *t*. When the drop starts to spin, a conspicuous sizzle emerges and lasts throughout the spinning process.

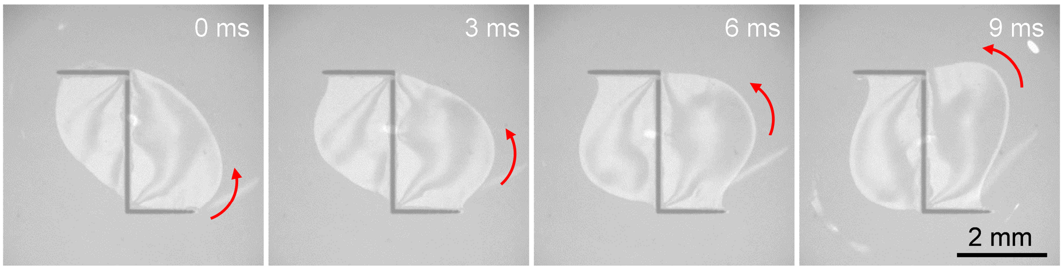

Supplementary Figure 5. Two vapour flows under the drop. The flow direction is consistent with the spin direction.

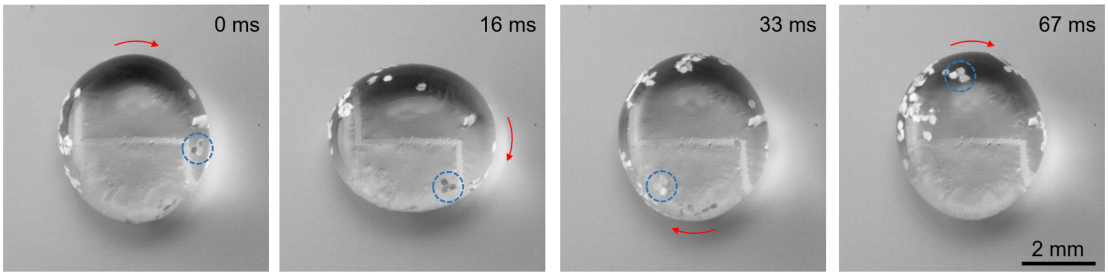

Supplementary Figure 6. Velocity gradient exiting in the drop. Plastic particles with a radius of 70 μm are placed in a rotating drop. the particles floating on the drop rotate 3/4 round when the drop makes a round. The red arrows indicate the rotation direction of the drop, and the blue dashed lines show the position of the particle.

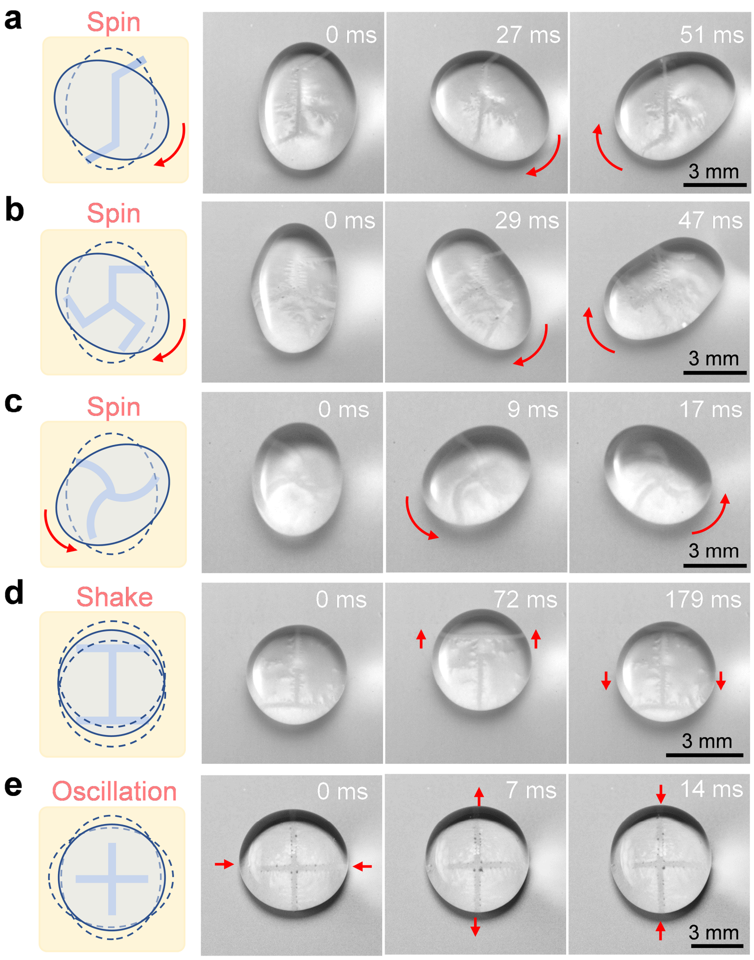

Supplementary Figure 7. Drops behaviors on expanded patterns. a, Drop spins on a 120°-angle pattern. **b**, Drop spins on a three-angle pattern. **c**, Drop spins on a three-arcs pattern. **d**, Drop shakes on an H-shaped pattern. **e**, Drop oscillates on a cross-shaped pattern.

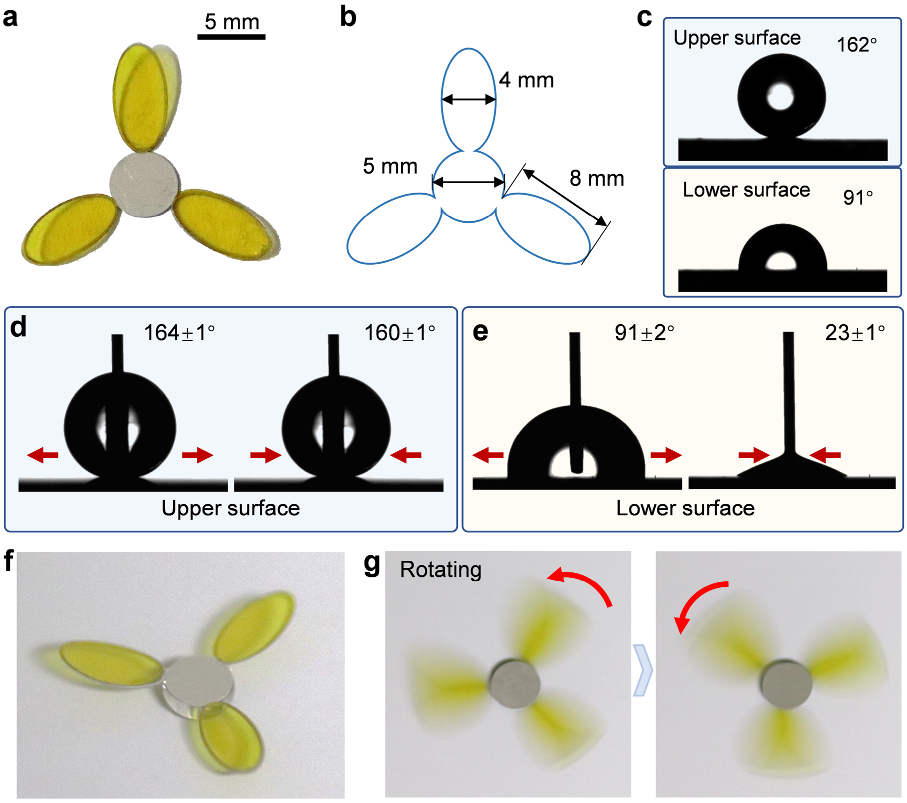

Supplementary Figure 8. Simplified droplet steam engine. a, Photograph of the airscrew. **b**, Parameters of the airscrew. **c**, Contact angles of the upper and lower surfaces of the rotor. **d** and **e**, Advancing contact angles and receding contact angles of the upper and lower surfaces of the rotor. **f**, Image of the airscrew sitting on a drop. **g**, Heating the surface resulting in the spinning of the airscrew.

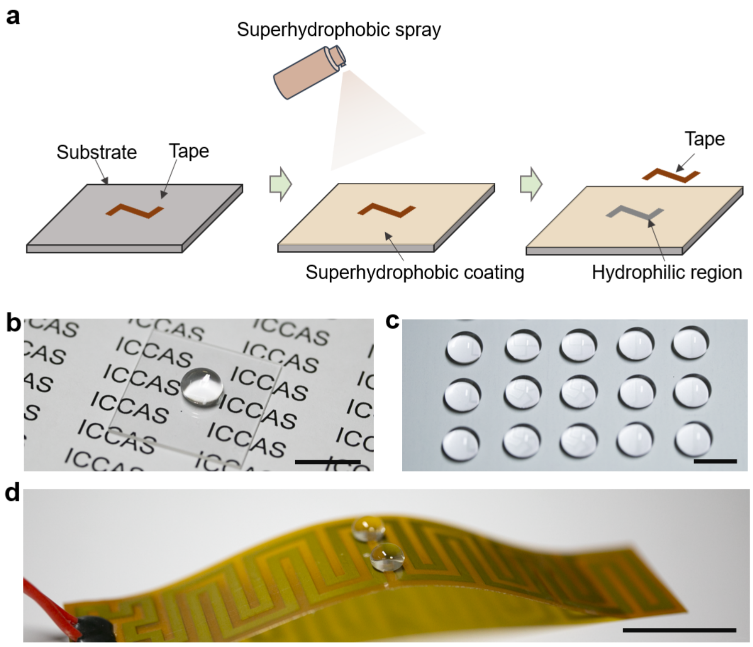

Supplementary Figure 9. **a**, Preparation of the wettability pattern by a spraying method. **b-d**, Demonstrations of drops placed on the glass, metal foil, and plastic film with wettability patterns that are prepared by the spraying method. Scale bars in **b** and **c** are 5 mm, and in **d** is 10 mm.

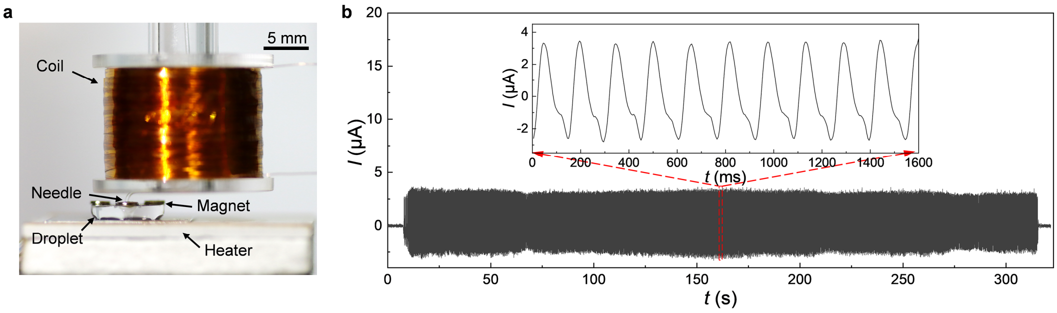

Supplementary Figure 10. Droplet electricity generator. The spinning drop can actuate magnets, converting the mechanical energy into electricity. **a**, Photograph of the drop electricity generator. An aluminum sheet equipped with three magnets is placed on a heated drop. A solenoid coil is fixed above the magnets to generate electricity. For continuous rotation, a needle connected with a syringe pump is used to supplement water into the drop. The output current I within 300 s is shown in **b**.

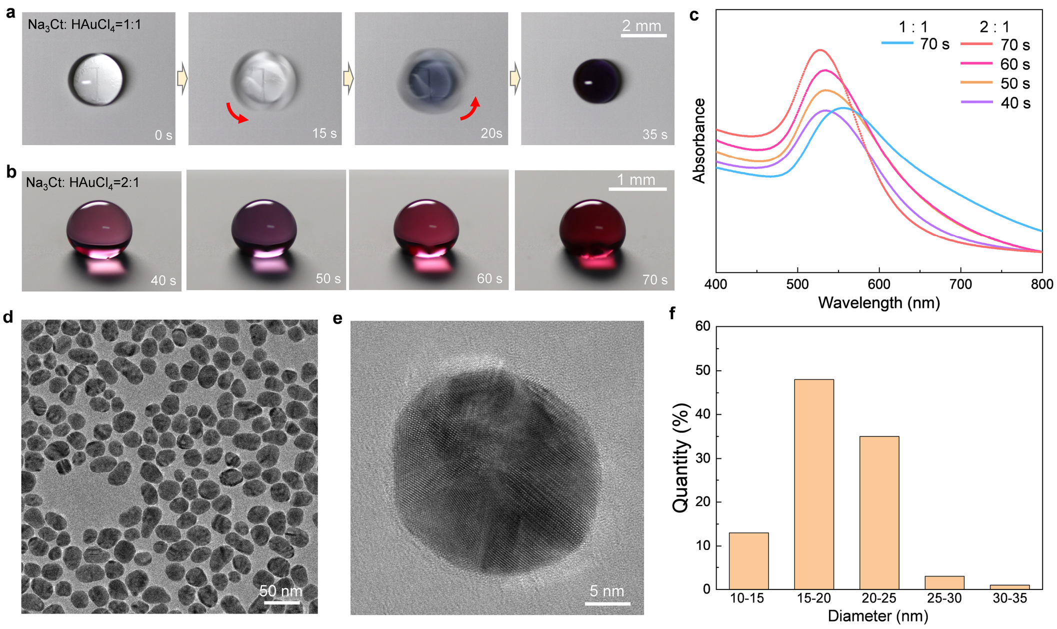

Supplementary Figure 11. Synthesis of gold nanoparticles using the drop gyro as microreactors. a, Evolution of a drop of precursor solution on the hot patterned substrate. The precursor solution contains sodium citrate and auric acid with a mole ratio of 1:1. The color of the drop gradually changes from colorless to dark blue. **b**, Altering the mole ratio of the reactants and controlling the reaction time can be employed to adjust the gold nanoparticles size. Scale bar, 1 mm. **c**, UV-vis spectra of the gold nanoparticles solutions synthesized in **a** and **b**. **d**, Transmission electron microscope (TEM) image of the gold nanoparticles. Scale bar, 100 nm. **e**, High-resolution TEM image of a single gold nanoparticle. Scale bar, 10 nm. **f**, Particle size distribution of the synthesized nanoparticles.

2. Supplementary Text

2.1 Discussion of the phase diagram

As shown in Fig. 1f, the drop spin mainly occurs between 115°C and 135°C. At a low temperature, the drop slightly shakes on the patterned wettability surface; by contrast, the drop vigorously detaches from the pattern at a high temperature. Meanwhile, the volume of the drop also affects the spin behaviours.

To achieve steady drop spin, two conditions need to be met: (1) the driving force (the viscous force from the vapour to the drop) should be larger than the resisting force (the adhesion force between the drop and the substrate); (2) the superhydrophilic pattern needs to seize the spinning drop.

When the temperature is lower than 115 °C, the continuous vapour film cannot form and there exists a large resisting force (the adhesion force) from the superhydrophobic region; besides, the driving force (the viscous force) is relatively small due to the weak vaporization and vapour flow. As a result, the drop cannot spin but slightly shake in this situation.

With the increase of the substrate temperature (115 °C and 120 °C), the resisting force (the adhesion force) decreases because of shrinkage of the contacted area due to the formation of the vapour film between the drop and the substrate, while the driving force (the viscous force) increases since the enhanced vaporization and the accelerated vapour flow. Once the driving force is larger than the resisting force, the drop begins to spin. In this temperature range, the drops smaller than 40 μl cannot spin because their sizes are smaller than the superhydrophilic pattern.

Further increase of the substrate temperature (125 °C to 135 °C) results in more violent

vaporization in the superhydrophilic pattern, which leads to the increase of the vapour pressure beneath the drop and the decrease of the capillary force between the drop and the superhydrophilic pattern. The spinning drop will detach from the superhydrophilic pattern if the pattern cannot seize the drop. In this temperature range, the drop spin only occurs at a moderate volume. This may be because large drops have large rotatory inertia, while small drops have small gravity (less than the vapour pressure at the drop bottom). In these conditions, the superhydrophilic pattern cannot seize the drops.

When the temperature of the substrate is above 135 °C, the vaporization at the superhydrophilic pattern is extremely violent, and the superhydrophilic pattern cannot seize the drop. Thus, the drop detaches from the superhydrophilic pattern in this temperature range.

2.2 Driving force of the drop spin

We utilized a model to quantitatively understand the drop spin, as well as the relationship between the rotational speed and the drop radius. Fig. 2d describe the parameters of the superhydrophilic pattern and the levitated drop, where h is the thickness of the vapour film beneath the drop, R is the equatorial radius of the drop, l and d are the width and the side length of the superhydrophilic pattern.

The mass change of the drop on the heated substrate with a wettability pattern is shown in Fig. 2e, which demonstrates a drop evaporation rate of about 1.2 mg s^{-1} during the process of drop rotation (29 s to 112 s). This value is much larger than the evaporation rate in previous studies, that is:

$$\dot{m} = \frac{k \Delta T l d}{L \delta_{\mathrm{t}}} \tag{1}$$

where k, L, and ΔT are the thermal conductivity, the vaporization latent heat of water, and the

temperature difference between the drop and the substrate (20 °C). Considering R = 2 mm, Equation 1 yields $\dot{m} \approx$ 0.1 mg s^{-1}. This is probably because Equation 1 was based on the assumption of steady-state heat transfer with no boiling, whereas in reality a violent contact boiling occurs at the hydrophilic region under the drop.

Considering the vapor incompressible in our system, the vapor flow speed under the drop U can be obtained by the following equation:

$$\dot{m} = 2\pi R U h \rho_{\mathrm{v}} \quad (2)$$

where h is the thickness of the vapor layer, ρ_{v} is the density of the vapor.

The lubrication approximation equation links the pressure ΔP to U and h, that is:

$$\frac{\Delta P}{R} = 12\mu_v \frac{U}{h^2} \quad (3)$$

where μ_v is the viscosity of the vapor. For the drop with a radius of 2 mm, ΔP can be considered as the hydrostatic pressure under the drop, thus $\Delta P \approx 2\rho g l_c$. At 100°C, the capillary length of water l_c is about 2.5 mm, resulting in a pressure of 50 Pa under the drop.

Combining the above equations, we obtain U and h:

$$U = \frac{(\rho g l_c)^{1/3} \dot{m}^{2/3}}{2\pi R \mu_v^{1/3} \rho_v^{2/3}} \quad (4)$$

$$h = \left(\frac{\mu_v \dot{m}}{\rho g \rho_v l_c}\right)^{1/3} \quad (5)$$

Typical values for the parameters are: $\rho \approx 10^3$ kg m^{-3}, $g \approx 9.8$ m s^{-2}, $l_c \approx 2.5 \times 10^{-3}$, $\dot{m} \approx 1.2 \times 10^{-6}$ kg s^{-1}, $R \approx 2 \times 10^{-3}$ m, $\mu_{\mathrm{v}} \approx 10^{-5}$ Pa s, and $\rho_{\mathrm{v}} \approx 1$ kg m^{-3}. Hence, we find h = 8×10^{-5} m, U = 1.2 m/s.

Then, we can calculate the driving force for the rotation, that is:

$$F_{\mathrm{d}} \approx \mu_v \frac{U}{h} \times d \cdot (2d) = \frac{(\mu_{\mathrm{v}} \dot{m})^{1/3} (\rho g l_c)^{2/3}}{\pi \rho_{\mathrm{v}}^{1/3} R} d^2 \quad (6)$$

We can simplify the calculation by assuming that the driving force acts at the corners of the

pattern. Hence, the driving torque M_d is:

$$M_{\mathrm{d}} \approx 2\frac{d}{\csc\pi/2}F_d \quad (7)$$

We find $F_{\mathrm{d}} \approx 7\times10^{-7}$ N, and $M_{\mathrm{d}} \approx 1.5\times10^{-9}$ Nm with d = 1.5 mm. The results are consistent with the previous studies of Leidenfrost drops driven on ratchet surfaces.

2.3 Resisting force of the drop spin

The spinning drop is subject to a resisting force. Three major forces are usually considered as the resisting force of the drop movement, including the adhesion force from the substrate, the air drag, and the viscous shear force. For the spinning drop, the adhesion force can be neglected, since the drop is levitated on the vapour layer and no direct contact exists between the drop and the superhydrophobic region.

Another possible resisting force is the air drag. For the drop with a radius of 3 mm and a rotational speed of 800 rpm, the linear velocity at the edge $U_{\mathrm{l}} \approx 0.3$ m s^{-1}. The Reynolds number in the air $Re = 2\rho_{\mathrm{v}}RU_{\mathrm{l}}/\mu_{\mathrm{v}}$ is at the scale of 10^2. Hence, a boundary layer develops around the drop with a characteristic thickness δ', and the resisting force of the drop movement is the viscous drag in the boundary layer. Since $\delta' \sim \sqrt{\nu_{\mathrm{v}}t}$, we can get this drag:

$$F_{\text{air drag}} \sim \sqrt{\rho_{\mathrm{v}}\mu_{\mathrm{v}}}(RU_{\mathrm{l}})^{3/2} \quad (8)$$

here $\nu_{\mathrm{v}} = \mu_{\mathrm{v}}/\rho_{\mathrm{v}}$ is the kinematic viscosity and t is the time of development of the boundary layer, naturally scales as R/U_{l}. With $R \approx 3$ mm, we find $F_{\text{air drag}}$ is at the scale of 10^{-7} N, much smaller than the driving force.

Two velocity gradients exist in the system that can generate viscous resisting force: the velocity gradient in the drop thickness direction and the velocity gradient in the liquid bridge. The resisting troque M_r is:

$$M_r = M_{r1} + M_{r2} \tag{15}$$

where M_{r1} is the resisting torque inside the drop and M_{r2} is the resisting torque inside the liquid bridge.

We have placed plastic particles with a radius of 70 μm on the surface of a rotating drop to roughly estimate the velocity gradient in the direction of drop thickness, as shown in Supplementary Figure 6. We find that the particles floating on the drop rotate 3/4 round when the drop makes a round, indicating a velocity gradient of $\omega/8l_c$ exists in the drop thickness. This gives the resisting torque generated inside the drop is:

$$M_{r1} \approx \oint_0^R \mu \frac{\pi\omega}{4l_c} r^3\, dr = \frac{\pi\mu\omega R^4}{16l_c} \tag{16}$$

We find M_{r1} is about 10^{-10} Nm with ω = 80 rad s^{-1}, R = 2 mm, which is much smaller than the driving torque.

In addition, the velocity gradient existing in the liquid bridge will also generate a resisting torque. Since the top of the liquid bridge connects to the rotating drop and the bottom of the bridge attaches to the substrate, a velocity gradient from 0 to ω exists over the thickness of the bridge, which equals the thickness of the vapor layer h. To simplify the calculation of the resisting torque, we consider the superhydrophilic pattern into a line and two arcs. Hence, the resisting torque M_{r2} is:

$$M_{r2} \approx 2\int_0^d \mu \frac{\omega r}{h} l \cdot r\, dr + 2\mu \frac{\omega l d^3}{h} = \frac{8}{3} \frac{\mu\omega l d^3 (\rho g l_c)^{1/3}}{(\mu_v \dot{m})^{1/3}} \tag{17}$$

We find this resisting torque is about 1.1×10^{-9} Nm, which is compared to the driving force. Therefore, we can consider that:

$$M_r \approx \frac{8}{3} \frac{\mu\omega l d^3 (\rho g l_c)^{1/3}}{(\mu_v \dot{m})^{1/3}} \tag{18}$$

2.4 Validation of the mechanical analysis

For a steadily spinning drop, the driving torque M_d should equal to the resisting torque M_r. Combining equations (6), (7), and (8) gives the rotating speed ω is:

$$\omega \sim a l^{-1} R^{-1} \tag{19}$$

where $a = (\mu_v \dot{m})^{2/3}(\rho g l_c)^{1/3}/6\mu\rho_v^{1/3}$. Typical values for the parameters are: $\mu_v \approx 10^{-5}$ Pa s, $\dot{m} \approx 1.2 \times 10^{-6}$ kg s^{-1}, $\rho \approx 10^3$ kg m^{-3}, $g \approx 9.8$ m s^{-2}, $l_c \approx 2.5 \times 10^{-3}$, $\rho_v \approx 1$ kg m^{-3}, $l \approx 10^{-4}$ m, and $R \approx 2 \times 10^{-3}$ m. We find $a \approx 2.5 \times 10^{-5}$ m^2, and ω is at the scale of 100 rad s^{-1}, which is consisting of the experimental data.

We plot the variation of the rotational speed ω versus the equatorial radius R in Fig. 2f. Drop spinning tests are performed on heterogeneous surfaces with superhydrophilic patterns of different widths. The results are demonstrated by the different colored dots. The rotational speed ω varies linearly with R^{-1}, while for a fixed R^{-1}, ω decreases with the increase of the superhydrophilic pattern width l. For a drop with a radius of 2 mm, ω changes from 60 rad s^{-1} to 90 rad s^{-1} as l changes from 70 to 130 μm, a slightly smaller change than the theoretical result, which may be because $\dot{m}$ also enlarges as l increases.
