# Supplementary material for: Tailoring vapor film beneath a Leidenfrost drop
Source: Nat Commun. 2023 May 8;14:2646. doi: 10.1038/s41467-023-38366-z (PMC10167315; doi:10.1038/s41467-023-38366-z)
Supplement: Supplementary file 3 — Description of Additional Supplementary Files [file 41467_2023_38366_MOESM3_ESM.pdf]

File Name: Supplementary Movie 1.
Description: Drops evolution on heated superhydrophobic and patterned wettability substrates. Drops with a volume of 80 μl are placed on the superhydrophobic and the patterned wettability substrates. The temperature of the substrate is 120 °C. On the superhydrophobic substrate, the drop slightly shakes in the entire process. By comparison, the drop shakes and then spins on the patterned wettability substrate. The spinning drop exhibits an ellipsoidal shape because of the balance between surface tension and centrifugal force. The movies are recorded at 500 fps and played in real-time, 1/5, 1/10, and 1/25 of real-time.

File Name: Supplementary Movie 2.
Description: Shaking, spinning, and detaching of the drops at different temperatures. Drops are placed on the patterned wettability substrates at 90 °C, 120 °C, and 140 °C, respectively. The volume of the drops is 80 μl. The drop slightly shakes on the substrate at 90 °C, while the drop steadily spins at 120 °C. On the substrate of 140 °C, the drop spins irregularly and then detaches from the superhydrophilic pattern. The movies are recorded at 3000 fps and played in 1/30 of real-time.

File Name: Supplementary Movie 3.
Description: Oblique view and bottom view of drops at different stages. In the initial stage, the drop slightly shakes on the patterned wettability substrate. The bottom view demonstrates that air bubbles (the light gray areas) exist around the superhydrophilic pattern (the dark grey areas). In stage II, the drop starts to spin, and a complete vapour film forms between the drop and the sapphire plate, except for the superhydrophilic pattern. In stage III, the drop spins rapidly and the shape changes from spherical to ellipsoid. The bottom view shows that the vapour film changes into an ellipse. The superhydrophilic pattern is constantly wetted by the drop. Besides, two vapour jets in accordance with the direction of the spin are clearly observed. The volume of the drop is 80 μl. The movie is recorded at 3000 fps and played in 1/100 of real-time.

File Name: Supplementary Movie 4.
Description: Infrared images of droplets of different sizes on a heated surface. Drops with a radius of 3 mm and 1 mm are placed on superhydrophobic substrates at 120 °C. A temperature gradient is obviously present in the 3 mm radius drop, while the 1 mm radius droplet does not have an apparent temperature difference. The movie is recorded and played in real-time.

File Name: Supplementary Movie 5.
Description: A droplet of 1 mm radius spinning on a patterned wettability substrate. When placed on a surface at a temperature of 120 degrees Celsius, a droplet with a radius of 1 mm begin to rotate. The movie is recorded at 3000 fps and played in 1/100 of real-time.

File Name: Supplementary Movie 6.

Description: Behaviours of drops on substrates with patterns of different symmetries. The drop spins on the Z-shaped pattern with a central symmetric. Using a superhydrophilic angle, the drop elongates and drifts toward the inside of the angle. On a stripe-shaped pattern, the drop stretches bilaterally and symmetrically, while unstable spinning occurs when placing a drop on a crowbar-shaped pattern. The temperature of the surfaces is 120°C. The movie is played in 1/30 of real-time.

File Name: Supplementary Movie 7.
Description: Droplet steam engine. The droplet steam engine consists of a patterned wettability surface, a gear, and a force transmission device to connect the drop and the gear. The diameter and height of the drop steam engine are 10 mm and 12 mm, respectively. After injecting 100 μl water, the drop can actuate the gear to rotate continuously for more than 40 s, with the maximum speed exceeding 840 rpm. The movie is played in real-time.

File Name: Supplementary Movie 8.
Description: Simplified steam engine. A rotor with three wings can steadily float on the drop through surface tension. When the drop spins, the adhesion force between the drop and the rotor actuates the rotor. The rotational speed of the rotor increases with time, with the maximum rotational speed exceeding 900 rpm. The rotor can steadily rotate for 50 s. The movie is played in real-time.

File Name: Supplementary Movie 9.
Description: Shaft driving using a wettability pattern in limited spaces. When water is injected into the narrow gap, the wettability pattern regulates the water vapour, actuating the drop rotates to drive the upper shaft. The temperature of the lower pillar is 130 °C. The distance between the shaft and the pillar is 1 mm. The movie is played in real-time.

File Name: Supplementary Movie 10.
Description: Directional transport of a drop on a patterned wettability surface. After placing on a surface with a fishbone-shaped pattern, the drops move in the direction of the pattern opening. The temperature of the substrate is 135 °C. The movie is recorded at 1000 fps and played in 1/4 of real-time.

File Name: Supplementary Movie 11.
Description: Directional movement of a drop on an inclined patterned wettability surface. Tilting the surface with the fishbone-shaped pattern, the drop can still move in the direction of the pattern opening (the left side). The substrate is tilted at an angle of 5° and the temperature is 135 °C. The movie is recorded at 1000 fps and played in 1/4 of real-time.
